# The Profession and Our Dental Colleges

**Published:** 1895-12

**Authors:** Truman W. Brophy

**Affiliations:** Chicago, Ill.


THE
AMERICAN JOURNAL
—OF—
DENTAL SCIENCE.

VOL. XXIX. BALTIMORE, DECEMBER, 1895. NO. 8.

ARTICLE I.

### THE PROFESSION AND OUR DENTAL COLLEGES.

BY DR. TRUMAN W. BROPHY, CHICAGO, ILL.

Read before the Illinois Society.

The dental colleges of the United States, by concert of action, have advanced the curriculum, so that it is today more systematic than in other professional schools.

The effort of the National Association of Dental Faculties to unify the course of study in the several institutions, while not entirely successful, has brought the colleges closer together in the methods of teaching, and the matter taught, than prevailed before the organization of that body.

The extension of the course of study over a period of three years has made it possible for students to bestow more time on the practical phases of anatomy, histology, bacteriology, chemistry, technology. etc., besides becoming more proficient in operative and prosthetic dentistry. The changing of the course, therefore, from two to three years has not only met with the approval of the profession, but has been equally gratifying to the faculties; so this relation of our colleges to the profession has been most happy.

"It will not be doubted by any one that it is our solemn duty in the conduct of dental education to see to it that before a student is permitted to enter on its study he has shown prompt aptitude and has already acquired certain fundamental knowledge. It avails nothing to point to the rare illustrious men who have leaped at a few bounds from the plowshare to the front rank of scientific or judicial eminence, and who have served immemorially as instances to support the fallacies of those who from selfish motives or from ignorant prejudice decry thorough education. Let not the rest of us, only ordinary sons of toil, be misled by the hope of such exceptional careers."

The relation of our colleges to the profession in regard to making our students more proficient practitioners scientifically, as well as practically, is one on which the colleges and the profession are interdependent. A practitioner may send to a college a student whose educational qualifications for admission are not of as high an order as they should be, and consequently his path to success is a most laborious one.

I cannot better express my ideas on the subject of preliminary education than to quote from Mr. I. Ernest Lane, Fellow of the Royal College of Surgeons, England, who, in an article on medical education and medical practice,* states that too much stress is laid on the benefits to be derived from what is termed a modern education, and suggests that a judicious admixture of the ancient with the modern would be of more benefit than an exclusive knowledge of either the one or the other. He points out that anatomical nomenclature, and in fact nearly all the technical terms met with in the study of medicine, are derived either from Latin or Greek, and that it is necessary to acquire at least an elementary knowledge of these languages in order to obtain an intelligent knowledge of their profession. As bearing on this subject, he quotes the words of a distinguished member of the profession, who

*British Medical Journal, Oct. 7th, 1893.

had recently given it as his opinion that, "if a boy destined for a profession, were to be taught nothing but reading, writing and arithmetic till he was eleven, and after that nothing but Greek, Latin and mathematics till he was eighteen, at fifty that boy would turn out a more widely cultured, better read man than if in his early years he had been stuffed with geography, history, philosophy and the two-penny halp-penny fragments of chemistry, botany and zoology which constitute school science."

If the views of Mr. Dane were presented to educators of large experience in medical and dental schools, I have no doubt that, after deliberate consideration, they would be heartily endorsed.

As we look over the directory of American dentists and place a mark opposite the names of those whose writings have made an indelible impression on our literature, and a profound impression on the dental mind, we note how few have enjoyed the great privilege of a university education. Had such opportunities been offered and embraced, however, the sphere of usefulness of these leaders in dentistry doubtless would have been materially enlarged.

On the other hand, a practitioner places a student in college whose affluent circumstances and broad preliminary education seem to especially fit him for his long course of professional study. His funds enable him to live comfortably, his education renders his professional study comparatively easy, but is there not another qualification essential to the success of a dentist?

It is my opinion that a course in a manual training school and a certificate of satisfactory work from teachers in such an institution, would be a very important stepping stone in entering the dental profession. Such a preliminary course prepares the student to engage in the manipulation of metals, the handling of fine instruments, and the manual dexterity essential to his success as a dental practitioner. A thorough knowledge of anatomy, chemistry, physiology, histology, materia medica and therapeutics, bacteriology, pathology and surgery, indispensible as it is in rendering a man qualified to practice dentistry, would very poorly indeed prepare him for the duties of his profession if he had not the manual dexterity to skillfully practice operative and prosthetic dentistry. My experience as a teacher—not extending over many years—has led me to the belief that all colleges should reserve the right to return to the student his fees and dismiss him whenever, in the judgment of the faculty, he cannot acquire the manipulative skill essential to enable him to practice dentistry successfully. To retain a student who by nature cannot acquire such skill and carry him on through a three years' course of study, and then reject him at the close of his final examinations, seems too unjust on the part of an institution to be excused.

Again, I think a student should never attempt any pursuit, unless he has a special desire to make it a life work or his vocation. I have no faith in the success of an individual who will take up the study of a profession just for a makeshift, or for what money he may possibly see in it. His work must be pursued with that interest that comes with a love for it, and he must be prompted by the desire to excel, not stand among the mediocrity of his profession, but with a determination to reach the topmast rung of the ladder of professional fame. Opportunity, it is true, may have a great deal to do with that, but a man's will power has a great deal more. A student who takes up the study of dentistry should be impressed by his preceptor with the fact that he has undertaken one of the most confining, laborious and enervating occupations known. It is only by force of habit that the busy dentist is able to get the outdoor exercise necessary to preserve his health.

The necessity of imparting practical instruction to students makes it necessary to have a well-equipped infirmary in every college, and patients on whom all of the operations in dentistry are to be performed. The question of how to supply students with patients has taxed the faculty of every dental college when first organized; and then the profession have in many instances criticized colleges for accepting patients who were not in a financial condition to warrant their applying to a college infirmary for operations. It seems that a matter of this kind should be so adjusted between the members of the profession and the colleges as to be quite satisfactory. It is true that dental college infirmaries often receive and perform operations for persons who are abundantly able to compensate a practitioner for his services, but the question of the patient's financial standing cannot be easily determined by college authorities. On the other hand, it is equally just to say that members of the profession extract many teeth which might be filled with cement or amalgam in a college infirmary for a fee which the patient might well afford to pay. Many such teeth are extracted because the patient would not be able to pay a fee for filling them that would compensate a dentist for his services. Therefore, it seems to me that poor patients, who have their teeth extracted for want of money to have them treated and filled should, instead of having them extracted, receive a certificate to a college infirmary, issued by the dentist, stating that this patient's circumstances are such that he should be admitted to an infirmary and receive such treatment as is necessary.

This brings us to the question of the relation of the profession to our dental colleges. The colleges must have patients, that they may teach practical dentistry. The colleges should not receive patients who are financially able to compensate dentists for their services. Members of the profession should not extract teeth which are susceptible of being treated and filled, on the ground that the financial condition of the patient will not permit them to spend the necessary time to treat them and put them in a healthy, satisfactory condition, but should, in all such cases, issue a card to such a patient and send him to a college infirmary, where such a card should admit him to treatment.

I believe not more than 20 per cent. of the population of the United States employ dentists for the purpose of treating and preserving their teeth. The remaining 80 per cent. do not engage the services of a dentist for any other purpose than for the extraction of teeth and the insertion of artificial ones. I do not make this statement from gathered statistics, but as an estimate made in a general way. Let us assume that there are 800 dentists in the city of Chicago, with a population, in all probability, of at least 2,000,000 of people, and each dentist treats and fills teeth for 500 people during a year. This would make 400,000, or 20 per cent. of the entire population of the city, the remaining 1,600,000, if this statement be correct, would employ dentists only for the extraction of teeth and making of plates. It seems unnecessary to state to this Society that the work devolving on us, and especially to the residents of Chicago, in educating this 1,600,000 people up to the value of the teeth and the advantages to be derived by preserving them, is a greater work than the most earnest educator has contemplated.

The mission of the dental college has long been supposed to be for the preparation of young men to engage in the practice of dentistry. Its teachings have elevated dentistry and placed it on a higher plane than it otherwise could have taken, but its usefulness is by no means limited to the preparation of students for dental practice. In my opinion its most important work is in educating the public to an appreciation of the great benefits which are bestowed on them by scientific and practical dentistry.

Many a young man is quite willing to assert that the nucleus of his practice was the patient whom he first met in a dental college infirmary.

The law governing the practice of dentistry in the State of Illinois has accomplished a great deal for the profession and for the public. The benefits that the profession have derived have been in a realization that men entering on practice in our State have been required to present credentials authorizing them to practice or take a State Board examination. It is true that our law has certain defects which should be amended; and in speaking on this subject I feel that it comes within the province of my paper, for it is the relation of the profession to the schools from which our ranks are augmented. I believe that the Illinois State Board of Dental Examiners has endeavored, from the time that the Board was first organized up to the present, to conscientiously discharge its duties. I am also aware that it is impossible for a body of men acting in that capacity to please every one, and what I shall say in regard to the relation of the Board to colleges and to the profession is not said in a spirit of fault-finding. It is not with a view of hurting the feelings of any one; but certain facts confront us, and it seems that steps should be taken to correct what is generally regarded as an error. The National Association of Dental Faculties, by increasing the course of college study from two to three years, did so that young men might become more proficient in dental knowledge. It seems, therefore, that the portals of the profession should not be entered by a shorter path. It has transpired on several occasions that permanent licenses have been issued by our State Board to young men who have had but a single course of study; and so their further study has been discontinued, and further proficiency has not been acquired. I do not believe it is the desire of the State Board of Dental Examiners to lower the standard of the profession by admitting to our ranks men who should pursue their studies further and complete their professional education. The relation which the profession should bear to colleges should be to aid them by counsel and friendly intercourse in the discharge of their momentous duty, and send to them only such young men as are qualified by nature and by education to intelligently pursue the study of dentistry. The practitioner's further duty is to support the infirmary, as far as can be, by sending patients to it who are unable to satisfactorily compensate him for his services, so that students may have an opportunity to acquire practical knowledge and that "delicacy of manipulation which comes only with experience."

Another suggestion I might make to the profession is to visit the dental colleges more frequently, and by their presence stimulate the students to make their very best efforts.

The duty of the colleges to the profession should be to guard their entrances and their exits; admit only such young men as possess a good education; watch them carefully; advance them practically and scientifically; and in the event of their failure to become skilled in the operating room and laboratories, they should reserve the right to return them their fees *pro rata*, and advise them to engage in some other vocation.

Another duty of the colleges to the profession should be to require certificates from persons whose financial ability to compensate a dentist for his services is at all questionable, and on such certificates admit them to the privileges of the college infirmary.

So, then, let the State Board of Dental Examiners, the National Association of Dental Faculties, the profession and the colleges, labor unitedly to advance our profession, so that our unprecedented achievements during the quarter of a century just closed may be rendered incomparable with those yet to come.—*Items of Intercst.*

